# Rapid Calorimetric Detection of Bacterial Contamination: Influence of the Cultivation Technique

**DOI:** 10.3389/fmicb.2019.02530

**Published:** 2019-11-01

**Authors:** Christian Fricke, Hauke Harms, Thomas Maskow

**Affiliations:** Department of Environmental Microbiology, Helmholtz Centre for Environmental Research – UFZ, Leipzig, Germany

**Keywords:** bacterial contamination, calorimetric detection, cultivation techniques, isothermal microcalorimetry, *Lactobacillus plantarum*, real-time monitoring

## Abstract

Modern isothermal microcalorimeters (IMC) are able to detect the metabolic heat of bacteria with an accuracy sufficient to recognize even the smallest traces of bacterial contamination of water, food, and medical samples. The modern IMC techniques are often superior to conventional detection methods in terms of the detection time, reliability, labor, and technical effort. What is missing is a systematic analysis of the influence of the cultivation conditions on calorimetric detection. For the acceptance of IMC techniques, it is advantageous if already standardized cultivation techniques can be combined with calorimetry. Here we performed such a systematic analysis using *Lactobacillus plantarum* as a model bacterium. Independent of the cultivation techniques, IMC detections were much faster for high bacterial concentrations (>10^2^ CFU⋅mL^–1^) than visual detections. At low bacterial concentrations (<10^2^ CFU⋅mL^–1^), detection times were approximately the same. Our data demonstrate that all kinds of traditional cultivation techniques like growth on agar (GOA) or in agar (GIA), in liquid media (GL) or on agar after enrichment via membrane filtration (GF) can be combined with IMC. The order of the detection times was GF < GIA ≈ GL ≈ GOA. The observed linear relationship between the calorimetric detection times and the initial bacterial concentrations can be used to quantify the bacterial contamination. Further investigations regarding the correlation between the filling level (in mm) of the calorimetric vessel and the specific maximum heat flow (in μW⋅g^–1^) illustrated two completely different results for liquid and solid media. Due to the better availability of substrates and the homogeneous distribution of bacteria growing in a liquid medium, the volume-related maximum heat flow was independent on the filling level of the calorimetric vessels. However, in a solid medium, the volume-related maximum heat flow approached a threshold and achieved a maximum at low filling levels. This fundamentally different behavior can be explained by the spatial limitation of the growth of bacterial colonies and the reduced substrate supply due to diffusion.

## Introduction

The fast and reliable detection of microbial contamination in food (Wadsö and Galindo, 2009; Velusamy et al., 2010; Khalef et al., 2016), water (Maskow et al., 2012) and pharmaceuticals (Buckton et al., 1991; Vine and Bishop, 2005) is necessary to prevent disease outbreaks as well as to ensure high quality, safety, and purity of these products. At the moment, the gold standard for the detection of microbial contaminations is a visual inspection of colony growth in case of solid media or of turbidity in case of liquid media or after standardized cultivation on selective solid media. Colony-forming units (CFU) per mL sample are counted for quantification of the contamination. The basic assumption is that one colony is formed per bacterium in the sample. Since about 10^5^ bacteria are necessary to form a colony visible to the human eye (Maskow et al., 2012), extended incubation is required depending on the specific growth rate μ_max_ of the microorganism. For instance, the standard specification prescribes 48 h incubation for the detection of *Pseudomonas aeruginosa* (ISO 16266:2006): (Jami Al-Ahmadi and Zahmatkesh Roodsari, 2016), 96–120 h for *Salmonella* (ISO 6579-1:2017): (Jay et al., 2005), and 120–144 h for *Listeria* (ISO 11290-1:2017) (Donoso et al., 2017). The great advantage over more sophisticated, molecular biological techniques like quantitative polymerase chain reaction (qPCR) combined with fluorescence detection or microscopic monitoring of selectively labeling probes is the simple handling and interpretation of results (Sieuwerts et al., 2008; Hazan et al., 2012). Biosensors represent another large class of detection methods for bacteria (Wang and Salazar, 2016). For instance, conductometric measurements provide rapid and easy handling detection of bacteria (Velusamy et al., 2010). The problem of non-specificity is overcome by selective antibodies but the sample matrix has a more significant influence since interfering quantities can play an important role (Law et al., 2015).

Many detection protocols include the enrichment of the bacteria using membrane filtration processes and subsequent placing the membrane filter onto a selective solid medium (ISO 7704:1985) to ensure that sufficient bacteria form colonies (Taylor et al., 1953; Tsuneishi and Goetz, 1958). Another modification is selective chemical pre-treatment of the sample like, e.g., the acidic treatment for the detection of *Legionella* (Bopp et al., 1981) serving to reduce the growth of unwanted, interfering microorganisms (ISO 11731:2017).

As an alternative to growth on solid media, it is also possible to detect bacterial contamination upon cultivation in (selective) liquid media. Here, mostly optical turbidity measurement is used to quantify the growth of the contaminant (Koch, 1970). Obviously, inherently turbid samples cannot be measured by this method. In addition, turbidity can be affected by dead cells, by-products of microbial activity such as polymers (Clais et al., 2015) or by precipitation.

We propose to monitor growth calorimetrically in order to achieve faster, more reliable, on-line detection that can be combined with different common cultivation techniques. High-performance isothermal microcalorimeters (IMC) are able to quantify tiny amounts of heat in the range from a few nano- up to microwatts (Braissant et al., 2010a). IMC can thus be applied as a real-time detector for a variety of microbial contaminants because all living microorganisms dissipate parts of the Gibbs energy of the substrates they assimilated in the form of heat (Gram and Sogaard, 1985; Wadsö, 1995; Von Stockar and Liu, 1999; Trampuz et al., 2007a; Maskow et al., 2012). The application of IMC for the quantification of microbial contamination by various species is already described in the literature (Levin, 1977; Xie et al., 1995; Trampuz et al., 2007a, b; Braissant et al., 2010b; Bonkat et al., 2011; Ren et al., 2012; Gysin et al., 2018). Interestingly, almost exclusively liquid cultures were used for the quantification of the different bacteria in samples from different origins. The application of calorimetry to bacteria grown on solid agar media was only described by Braissant et al. (2010b) for the bacterial quantification of *Mycobacterium tuberculosis*. The application of membrane filtration for bacterial quantification using IMC has to our knowledge not yet been described. However, qualitative IMC monitoring of the growth of mycobacteria on nylon filters (Solokhina et al., 2017), as well as the qualitative sterility testing of membrane filters (Brueckner et al., 2017) and experiments with three different species on pure titanium disks placed on solid media (Astasov-Frauenhoffer et al., 2012, 2014) could be considered as preparatory work in that direction.

Although IMC is known as a non-specific detection method, an appropriate sample preparation (e.g., chemical treatment) and selective culture media can be used to allow only the growth of microorganisms of concern, whereas accompanying microbial community is suppressed (Bujalance et al., 2006; Veselá et al., 2019).

Our study aims at the systematic investigation of the combination of common cultivation techniques with calorimetric monitoring, using *Lactobacillus plantarum* as a model strain for anaerobic systems, for its potential to identify microbial contaminations faster and more reliably. Emphasis is put on the influence of the cultivation techniques on calorimetric monitoring.

## Materials and Methods

### Bacterial Strain, Medium, and Cultivation

*Lactobacillus plantarum* DSM 20205 (German Collection of Microorganisms and Cell Cultures, DSMZ, Braunschweig, Germany) was used for the calorimetric investigations. The strain was cultivated on MRS (DE MAN, ROGOSA, SHARPE) medium, which is composed of (in g⋅L^–1^): glucose (20), peptone (10), meat extract (10), yeast extract (5), K_2_HPO_4_ (2), NaCH_3_COO⋅3 H_2_O (5), (NH_4_)_2_HC_3_H_5_(COO)_3_ (2), MgSO_4_⋅7 H_2_O (0.2), MnSO_4_⋅4 H_2_O (0.05) as well as 1 mL⋅L^–1^ Tween 80. Agar (1.5%) was used to solidify the medium. The final pH value was set to 5.7–6.2 by H_2_SO_4_ (*c* = 1 mol⋅L^–1^). One colony of a pre-grown Petri dish was used for the liquid pre-cultures of *L*. *plantarum*, which were incubated overnight at 30.0 ± 0.2°C (Unihood 650, UniEquip, Leipzig, Germany). Cells were harvested immediately before the calorimetric experiments. The identity of the species was regularly checked by the morphology of the colonies and once by 16S ribosomal DNA analysis.

### IMC Measurement

The calorimetric measurements were performed in 4-mL glass ampoules in a high-performance IMC (Thermal Activity Monitor III, TA Instruments, New Castle, United States). The ampoules and caps were autoclaved at 121°C for 40 min and then filled with a defined volume of media. In the case of solid media, the ampoules were filled with warm (∼70°C), molten MRS-agar medium, closed and stored at 4°C. The prepared glass ampoules containing the solid media were cooled down to room temperature before the bacteria were added. After adding the bacteria, the glass ampoules were set into a pre-heating position (to reach thermal equilibrium) for 15 min. In the last preparation step, the glass ampoules were pushed into the measuring position and after further 45 min (to reach thermal equilibrium), the heat flow signal was recorded.

The calibration of the TAM III was performed by an electric gain calibration (Joule heating) after certain time intervals to ensure precise measurements. The gain calibration consisted of two steps. First, a stable signal in each measuring channel was awaited (slope of the signal < 250 nW⋅h^–1^). Second, a single pulse of 1 mW was generated for 30 s by an inbuilt electrical calibration heater. The gain and offset were calculated as a result of the instrument response. All measurements were performed at 37.0 ± 0.0001°C. The obtained data were analyzed using *OriginPro* 2018. In all cases, where the high criteria for signal stability could not be met, a linear baseline correction was performed (see Supplementary Material).

### Sample Preparation

An overnight pre-culture of *L*. *plantarum* was diluted to an OD_600_ ∼ 0.15–0.20 (U-2900 Spectrophotometer, Hitachi, Tokyo, Japan) and this solution was diluted in series (dilution factors: *f*_a_ = 10^4^–10^8^). Four different test series were performed: (i) GOA, (ii) GL, (iii) GF (Supplementary Material) and (iv) GIA. All concentration-dependent experiments were performed in 200 μL MRS-medium. As an exception, the GIA experiments were performed in 1000 μL MRS-medium. In order to determine the reproducibility of these experiments at varied cell concentrations, triple measurements of the respective test series were conducted.

For GOA, 10 μL of each dilution were added onto the agar surface. For GL, also 10 μL of each dilution was added to the broth. For GF, 1.8 mL of each dilution was filtrated and the sealed adapters, as well as the membrane filters, were purged with 400 μL MRS media. The used sealed adapters were made to measure and fit for a standard membrane filtration system (Sartorius AG, Göttingen, Germany). In these experiments, sterile membrane filters (pore size 0.45 μm, Cellulose nitrate, Sartorius AG, Göttingen, Germany) were prepared for subsequent membrane filtration under sterile conditions using an autoclaved punch with a diameter of 10 mm (Locheisen-Satz, BGS Technic KG, Wermelskirchen, Germany). For GIA, 10 μL of each dilution was added to molten MRS-agar (45–50°C) and carefully mixed. The homogenization succeded at these relatively low operating temperatures by using a low agar concentration of 1.5 (w/w)%. In addition to the concentration-dependent measurements, the dependency on the filling level in the ampoules (*n* = 2) and the reproducibility at a constant concentration (*n* = 5) were investigated. However, sample preparation remained the same in all cases.

**FIGURE 1:**
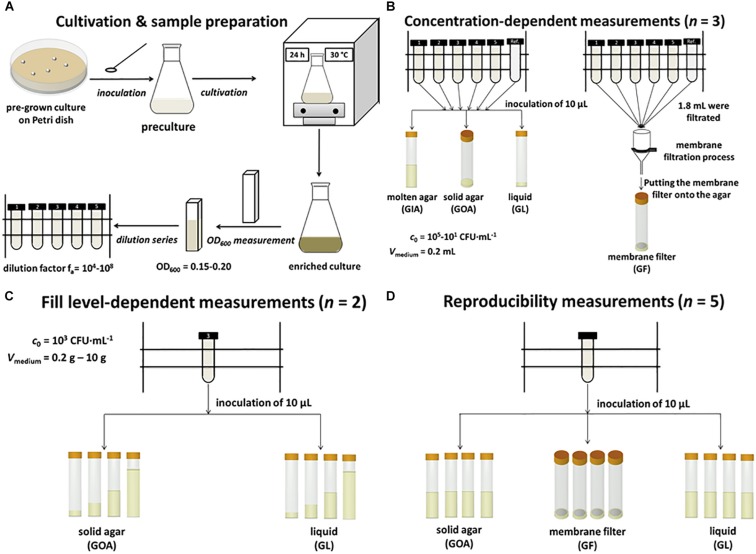
Schematic summary of the methodology. **(A)** Cultivation and sample preparation. **(B)** Concentration-dependent measurements of GIA, GOA, GL, and GF. **(C)** Fill level-dependent measurements of GOA and GL. **(D)** Reproducibility of measurements of GOA, GL, and GF.

Schematically, the individual sample preparation steps, as well as the cultivation, are summarized in Figure 1.

In order to quantify the initial concentration of bacteria, the colony-forming units (CFUs) per mL of each dilution step were determined in separate experiments (see Supplementary Material). For this purpose, each dilution step was plated in replicates on agar and incubated overnight at 37.0 ± 0.25°C (New Brunswick Innova 44, Eppendorf, Hamburg, Germany). In addition, the time required for a bacterial cell to form a visible colony was determined. After the first 11 h, the plates were hourly monitored and the colonies were counted after they became visible to the naked eye. To confirm this kind of experiments, a second experiment was performed by a fully automated monitoring process for all dilution stages under the same conditions. Every 10 min the agar plates were scanned and the resulting pictures saved as a jpg file. Subsequently, these files were evaluated. The same procedure was repeated with the growth of colonies on membrane filters. A short summary of these experiments is given in the Supplementary Material.

The reference measurements in liquid media (*n* = 9) were performed by monitoring the optical density in a 96-well plate by a photometer (Victor X3, PerkinElmer, Waltham, MA, United States) at 600 nm and 37.0 ± 0.1°C.

## Results and Discussion

### Theoretical Background

Establishing the relation between the earliest thermal detection time *t*_dect._ and the concentration of bacteria *N*_critical_ considered as critical requires a thermokinetic model that is as simple as possible. The simplest assumption is that each bacterium has the same heat production rate (Eq. 1) and that the growth dynamic of the bacterial population can be described by the Malthusian (Eq. 2) (Chang-Li et al., 1988).

(1) $$\phi \,\left(t\right)=N\,\left(t\right)\cdot \phi_{0}$$

(2) $$N\,\left(t\right)=N_{0}\cdot \exp\left({\text{ μ }}_{\text{max}}\cdot t\right)$$

Φ(*t*) (in W) and *N*(*t*) (in cells per L) are the heat production rate and the number of bacteria at time *t*, Φ_0_ is the thermal output of a single bacterial cell (in W per Cell). Typical values for bacteria (in the region of a few pW) in their dependency on growth conditions can be found for instance in Maskow et al. (2011), *N*_0_ is the initial concentration of bacteria and μ_max_ (in h^–1^) is the maximum specific growth rate. The earliest detection time of bacterial contamination obviously depends on the precision of the employed IMC. For instance, a heat flow precision of Φ = 2⋅10^–7^ W is described for the TAM III^1^. In reality, however, this thermal detection limit is seldom achieved due to thermal disturbances when the ampoule is inserted, traces of moisture on the outer shell of the glass ampoule, parasitic heat flows, etc. Assuming a detection threshold of Φ_dect._ = 2⋅10^–6^ W, the metabolically caused heat signal can be unambiguously differentiated from the noise of the microcalorimeter (Braissant et al., 2010b). Thus, it is now possible by combining Eqs. 1 and 2 to establish a simple linear correlation between the detection time *t*_*dect.*_ and the natural logarithm of the initial bacterial concentration *N*_*0*_ (Eq. 3).

(3) $$\ln N_{0}=-{\text{ μ }}_{\max}\cdot t_{\text{dect}.}+\ln\left(\frac{\phi_{\text{dect}.}}{\phi_{0}}\right)$$

The equation shows the two crucial adjustment screws for reducing the detection time (increasing the initial number of bacteria or reducing the detection limit of the calorimeter used). One might argue that the assumption of a cell-specific heat production rate is too unrealistic. However, if we drop this assumption and postulate more realistic that Φ_0_ depends on the growth rate, we come to a very similar correlation as before (Eq. 4) (Maskow et al., 2012).

(4) $$\ln N_{0}=-{\text{ μ }}_{\max}\cdot t_{\text{dect}.}+\ln\left(\frac{\Phi_{\text{dect}.}}{\mu_{\text{max}}\cdot \Delta_{\text{R}}\,H_{\text{X}}\cdot C_{c}}\right)$$

Here, Δ_R_*H*_X_ and *C*_*C*_ stand for the enthalpy change of growth reaction related to the formation of one C-mole biomass and the mean carbon content of a single bacterium, respectively. Independent of the applied equation, a linear relationship between initial concentrations of the bacteria and the detection time *t*_dect._ with μ_max_ as the slope is expected. With the aid of this relation, we can read off the corresponding detection time for any measured heat flow signal of a pure culture of *L*. *plantarum* and determine the associated initial concentration of bacteria. The final assumption needed for the practical application of this kind of quantification for microbial contamination is a constant specific growth rate μ_max_ during the considered detection times. These conditions are fulfilled when sufficient substrates are available and the bacteria are in the early exponential growth phase.

### IMC Measurements of the Different Cultivation Techniques

In the following, the application of the different cultivation techniques to IMC monitoring is described.

#### Growth on Agar (GOA)

**FIGURE 2:**
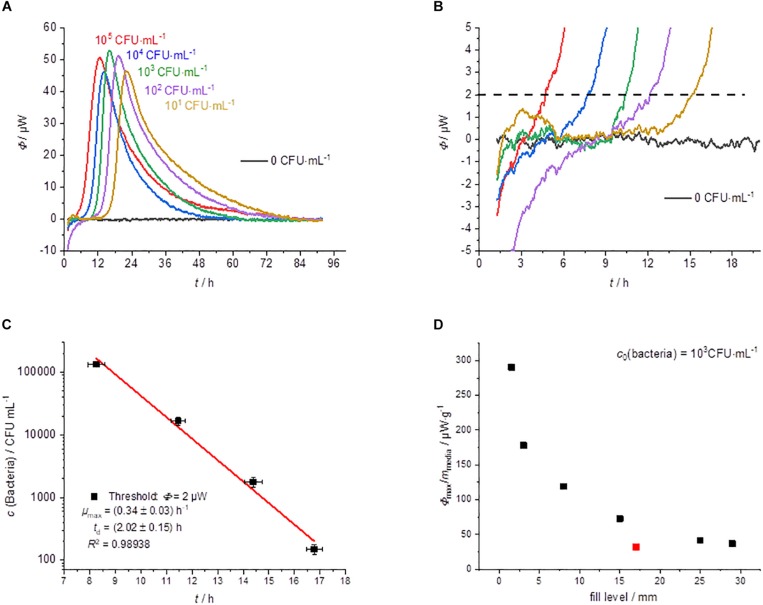
Summary of the IMC experiments performed on MRS-agar (GOA). **(A)** Dependency of the heat traces on the initial bacterial concentration. **(B)** Detail magnification of the heat signal near the quantification limit, the dotted line represents the postulated threshold value Φ_dect._ = 2 μW. **(C)** Dependency of the detection time on the initial bacterial concentration. **(D)** Specific maximum heat flow as a function of the filling level of the calorimetric vessel.

The results of the cultivation of *L*. *plantarum* on agar are summarized in Figure 2. *L*. *plantarum* can only ferment the substrate glucose, so we do not expect a complex heat flow signal as a result. The heat flow signals confirmed this expectation; only a single peak is observed in all measurements. After reaching a maximum the heat flow signal returned to the baseline. With decreasing initial concentration of the bacteria, the heat flow signal shifted as predicted by Eqs. 3 and 4 to later time (Figure 2A). Similar observations were also made by Braissant et al. (2010b) with the detection of mycobacteria. The threshold value for the corresponding detection time was as mentioned set to Φ_dect._ = 2 μW (Figure 2B).

Considering Eqs. 3 and 4 any other threshold value within the exponential growth phase should be resulting in the linearity of the logarithm of the initial bacterial concentration. This was already illustrated using heat flow curves of *Escherichia coli* (Maskow et al., 2012). Also in the present case, we obtained a linear relationship when plotting the detection times against the logarithms of the initial concentrations of bacteria (Figure 2C).

The results of CFU determinations are given in the Supplementary Material. For the highest concentration of bacteria, the detection took place after approximately 8 h and for the lowest concentration after approximately 17 h. Derived from the slope microbiological information like the specific growth rate μ_max_ was obtained. Table 1 summarize calorimetrically determined growth parameters and compare the calorimetric detection times of the different cultivation techniques.

**TABLE 1:** Summary of the results with different cultivation techniques combined with IMC experiments.

**Cultivation technique**	***V* (inoculum)/mL**	**μ_max_/h^–1^**	***t*_dect, high_/h**	***t*_dect, low_/h**
GOA	0.01	(0.34 ± 0.03)	8.3	16.8^b^
GL	0.01	(0.37 ± 0.01)	8.5	16.5^b^
GF	1.8	(0.33 ± 0.02)	4.7	15.9^c^
GIA	0.01	(0.40 ± 0.03)	8.9	13.6^d^
Reference (Wegkamp et al., 2010)	n.d.	(0.40 ± 0.03)^a^	n.d.	n.d.

*^*a*^*L*. *plantarum* WCFS1 growth on MRS-medium in 96-wells microtiter plates at 37°C; ^*b*^10^2^ CFU⋅mL^–1^; ^*c*^10 CFU⋅mL^–1^; ^*d*^10^3^ CFU⋅mL^–1^.*

If only cheaper and less accurate calorimeters are available, the loss of accuracy can be compensated by a larger filling level of the calorimetric vessel. However, the correlation between the filling level (in mm) and the corresponding metabolic heat flow must be known. Interestingly, the specific maximum heat flow signals (in μW⋅g^–1^) versus the filling level of the calorimetric vessel is approaching asymptotically a border value (Figure 2D). Small amounts of solid nutrient medium with a low filling level show a comparatively larger specific maximum heat flow. However, if the filling level increases, the specific maximum heat flow decreases and reaches a constant value of about 30 μW⋅g^–1^. This behavior seems to be independent of the applied calorimeter, because the red measuring point (Figure 2D), determined by a less powerful calorimeter (MC-Cal/100 P, C3 Prozess- und Analysetechnik GmbH, Munich, Germany) fits perfectly into the correlation determined with the high-performance calorimeter.

The asymptotic limit value approach during GOA-filled ampoules can be explained as follow. The maximum heat production is determined by the number of bacteria and their access to the substrate. The substrates are supplied by diffusion. At low fill levels, the diffusion paths are short and the number of bacteria produced depends on the absolute amount of substrate. At high fill levels, the maximum number of bacteria is limited by the surface of the agar and the delivery rates of the substrate are slow due to the long diffusion path. An improved surface to filling volume ratio and thereby a shorter diffusion pathway can be achieved by using slanted agar (Braissant et al., 2010b).

This GOA cultivation technique is preferably suitable for aerobic mesophiles as well as aerotolerant microorganisms such as our model strain. The method is also applicable to strict anaerobic microorganisms if oxygen is excluded during both preparation and the conduction of the experiments. This can be achieved by purging with an inert gas (e.g., N_2_) (Maskow and Babel, 2003) or by overcoating of the culture with paraffin oil as described by Xu et al. (2018).

#### Growth in Liquid (GL)

**FIGURE 3:**
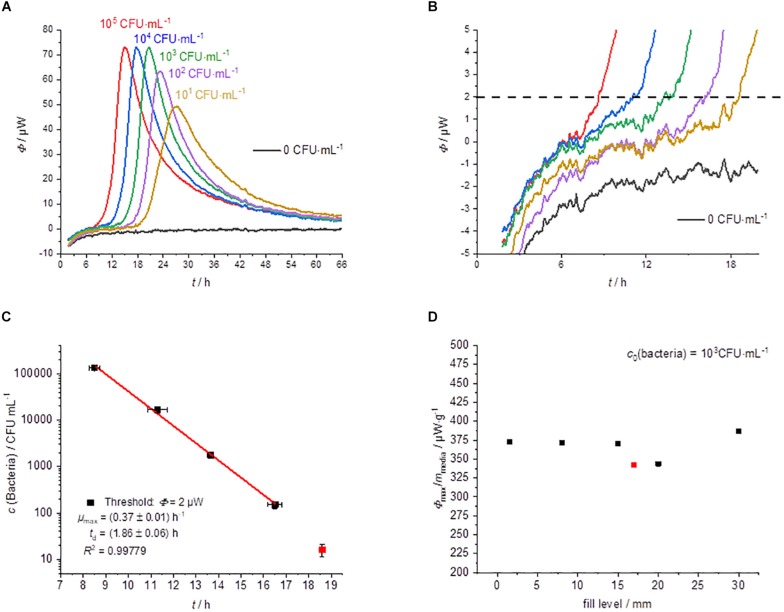
Summary of the IMC experiments performed in MRS-broth (GL). **(A)** Dependency of the heat traces on the initial bacterial concentration. **(B)** Detail magnification of the heat signal near the quantification limit, the dotted line represents the postulated threshold value Φ_dect._ = 2 μW. **(C)** Dependency of the detection time on the initial bacterial concentration. **(D)** Specific maximum heat flow as a function of the filling level of the calorimetric vessel.

The usual IMC approach is the cultivation in liquid medium (broth) (Boling et al., 1973; Trampuz et al., 2007b; Maskow et al., 2012; Braissant et al., 2015; Miyake et al., 2016; Dandé et al., 2018). The results with the liquid medium are summarized in Figure 3.

The shape of the heat flow signals is similar to that of the solid medium (Figure 3A). However, the standard deviation of the detection time is slightly smaller than for measurements on solid agar (Figure 3C). One reason might be the better homogeneity of bacteria and substrate in the liquid medium.

The detection times were between 8 h (for the highest concentration of bacteria) and 18.5 h (for the lowest concentration) using liquid media (Figure 3C). Garcia et al. (2017) were also able to find similar detection times for the microcalorimetric investigation of *Lactobacillus reuteri*. The lowest concentration (10 CFU⋅mL^–1^) was only detectable once of three measurements. At a concentration of 10 bacteria per mL, the usual inoculum of 10 μL contains statistically only one of a tenth of a bacterium. A comparison with the literature data shows that the specific growth rate (0.40 ± 0.03 h^–1^) (Wegkamp et al., 2010) measured for *L*. *plantarum* WCFS1 is very similar compared to our specific growth rate (0.37 ± 0.01 h^–1^). The obtained microbiological information from this experiment is summarized in Table 1.

In contrast to experiments on solid medium, the specific maximum heat flow is constant and independent of the filling volume (Figure 3D). This finding is confirmed by a further experiment using a less sensitive calorimeter (MC-Cal/100 P, C3 Prozess- und Analysetechnik GmbH, Munich, Germany) with different geometry of the calorimetric vessel and a filling volume of 10 mL (see the red measuring point in Figure 3D). Gysin et al. (2018) observed similar behavior for their sample volume optimization in the range from 0.25 to 2 mL. This is explainable because in liquid culture, the bacteria and the substrates are mainly homogeneously distributed and effects of the vessel size, therefore should not play a role.

#### Growth on Membrane Filter Onto Agar (GF)

**FIGURE 4:**
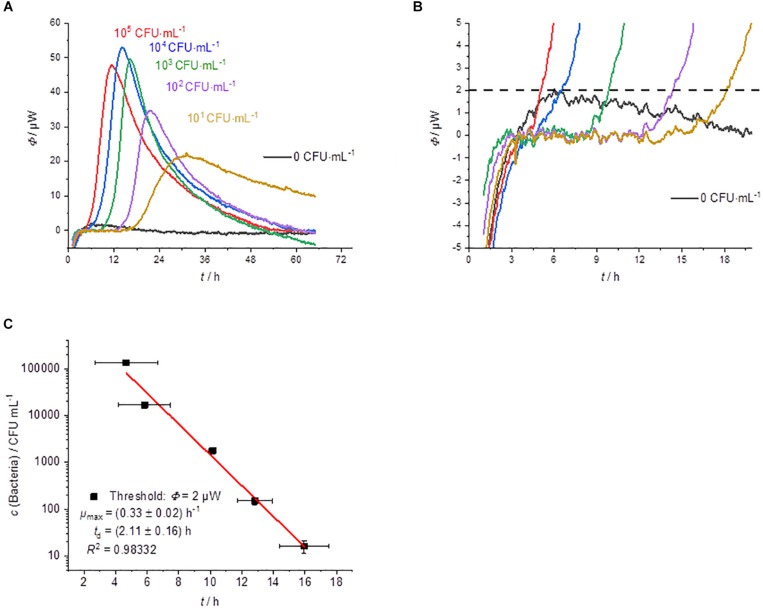
Summary of the results of the cultivation of membrane filters onto MRS-agar performed by IMC experiments (GF). **(A)** Dependency of the heat traces on the initial bacterial concentration. **(B)** Detail magnification of the heat signal near the quantification limit, the dotted line represents the postulated threshold value Φ_dect._ = 2 μW. **(C)** Dependency of the detection time on the initial bacterial concentration.

Figure 4 summarizes the results of the cultivation of membrane filter onto MRS-agar. The heat flow signals again follow a simple peak curve. However, the peak width is much wider than in the previous measurements. Due to the larger sample volume, which can be applied due to the membrane filtration step, the initial bacterial concentration is much higher than with the other techniques. As a result, the detection times are even shorter (Figures 4A,B).

As expected, the GF experiments also show a linear dependency between the detection time and the logarithm of the initial bacterial concentration (Figure 4C). The slightly lower correlation can be explained by the fact that the CFUs per mL were determined from the original samples. A better approach might be to use the counted CFU per mL directly from the membrane filtration since there might be differences in the growth of single colonies between plating and growth on membrane filters (Clark et al., 1951; Taylor et al., 1953).

Nevertheless, the specific growth rate and the doubling time of the bacteria fit to the previous GOA experiments. The bacterial growth is slightly slower compared to GL. For the highest concentration of bacteria, the detection takes place after approx. 4.5 h and for the lowest concentration after approx. 16 h. As already mentioned, the earlier detection is due to the larger volume (1.8 mL), which was filtered in these experiments. Measurements depending on the filling level of the calorimetric vessels were not performed due to the similarity to GOA experiments.

#### Growth in Agar (GIA)

**FIGURE 5:**
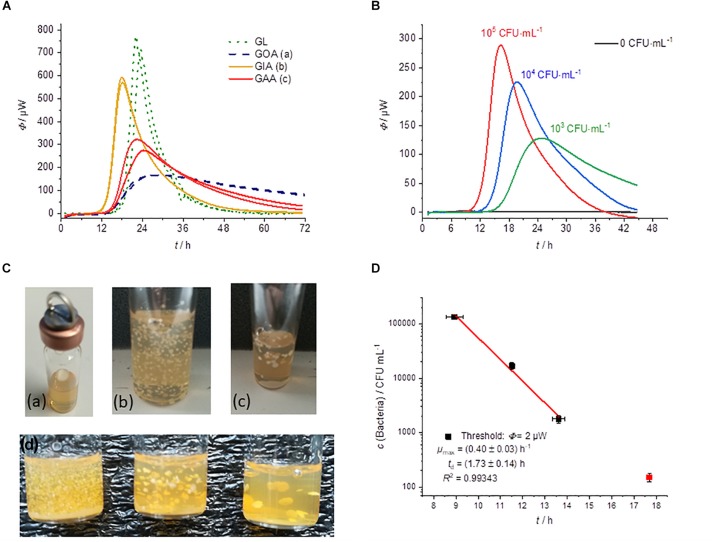
**(A)** Heat flow measurements of GL (green dotted), GOA (blue dashed), GIA (orange), and GAA (red). All experiments were performed in 2000 μL medium using an initial bacterial concentration of 10^3^ CFU⋅mL^–1^. **(B)** Dependency of the heat traces on the initial bacterial concentration for GIA experiments. **(C)** Photographs of different cultivation techniques with solid media: **(a)** GOA, **(b)** GIA, **(c)** GAA, **(d)** GIA of inocula with densities that decrease from left to right. **(D)** Dependency of the detection on the initial bacterial concentration for GIA.

In addition to the classical spreading (or plating) of bacteria, there is another approach: the so-called pour plate method (GIA). It is the prefered technique for counting CFUs per mL of bacteria in viscous fluids (Vansoestbergen and Lee, 1969). For IMC measurements it might also be the prefered technique for strict anaerobic as well as for microaerophilic microorganisms (if strong anaerobic conditions were not met). Figure 5 shows that GIA can be also combined with calorimetric monitoring.

Figure 5A compares the different cultivation techniques. Note, that in the case of GIA an inoculum volume of 100 μL was used. The mixing of inocula with the medium allows the using of larger sample size with a higher number of initial bacteria and thus a shorter detection time in comparison to the GOA approach.

Furthermore, with GIA higher heat flow signals in comparison of GOA can be observed, because the colonies are evenly distributed in three dimensions instead of being restricted to a surface (2D). Therefore, substrate availability is better for GIA. The homogeneous 3D-distribution can be seen in Figure 5C. In order to understand the observed effect better, we investigated a case between GIA and GOA. The bacteria were placed between two agar layers (GAA). The availability of the substrates should therefore also be better than in the case of GOA. The red heat flow curves in Figure 5A illustrates that due to the better substrate availability, the maximum heat flow is approx. twice as large as in GOA and half of that in GIA. The observations are very consistent with the IMC measurements of Kabanova et al. (2012, 2013). Figures 5B,C show the influence of the number on initial bacteria on the heat flow signal. In the case of a smaller initial number of bacteria, larger colonies were formed and the total heat flow was lower than for many bacteria (Figure 5B). At small bacterial concentrations, only one experiment was successful (red square, Figure 5D). We identified two potential reasons. Firstly, the inoculum volume (10 μL) might not have contained any bacterial cells. Secondly, the increased temperature of the still molten agar (∼50°C) might have killed part of the initial bacteria.

A comparison of the specific growth rates revealed that the growth rate of the bacteria did not depend on the physical state of the medium and the localization of the inoculum. Kabanova came to the same conclusion when they investigated *Lactococcus lactis* (Kabanova et al., 2013). The reason for this is that glucose has a similar diffusion coefficient in water as in solid agar medium (Schantz and Lauffer, 1962).

### Comparison With Conventional Techniques

A decisive question is what advantages (in terms of economy of time and accuracy) over conventional methods the IMC can achieve. In Table 2, IMC is compared with traditional counting and real-time measurement of the optical density.

**TABLE 2:** Comparison of the detection times (in h) determined by conventional techniques and IMC measurements.

**Measuring technique**	**10^5^ CFU⋅mL^–1^**	**10^4^ CFU⋅mL^–1^**	**10^3^ CFU⋅mL^–1^**	**10^2^ CFU⋅mL^–1^**	**10^1^ CFU⋅mL^–1^**
IMC – GOA	(8.3 ± 0.3)	(11.5 ± 0.3)	(14.4 ± 0.3)	(16.8 ± 0.3)	n.d.
IMC – GL	(8.5 ± 0.2)	(11.3 ± 0.4)	(13.7 ± 0.1)	(16.5 ± 0.3)	18.6^d^
IMC – GF	(4.7 ± 2.0)	(5.8 ± 1.6)	(10.2 ± 0.3)	(12.8 ± 1.1)	(15.9 ± 1.5)
Counting – GOA *t*_dect._^a^/h	13.7	13.7	15.7	15.7	17.7
*t*_dect._^b^/h	(15.8 ± 0.4)	(16.7 ± 0.6)	(17.0 ± 0.6)	(17.1 ± 0.5)	n.d.
OD_600_ – GL^c^	(13.1 ± 0.4)	(18.5 ± 1.3)	(24.3 ± 2.0)	(25.6 ± 3.1)	n.d.
Counting – GF^b^	11	12	15	17	18

*n.d., Not detectable. ^*a*^Detection time after colonies were countable by the eye – evaluation in real-time; ^*b*^Detection time after colonies were countable by the eye – evaluation of the scanning images; ^*c*^Only four of nine measurements could be evaluated; ^*d*^Less than three replicate heat flow signals were obtained, no standard deviation is indicated.*

If one first considers the high initial concentration (>10^2^ CFU⋅mL^–1^), the great advantage of the IMC measurement over the visual counting of individual colonies and monitoring of the optical density becomes apparent. The exact determination of such a high concentration as CFU is error-prone even with small inoculum volumes such as 10 μL because the colonies cannot be clearly separated (an intermediate dilution is necessary). In the case of IMC, every bacteria contributes to the heat and, therefore, high bacteria concentrations do not falsify the measurement results. In addition, bacterial growth can be detected 5–7 h earlier by IMC in comparison to counting. On the other hand, low initial bacterial concentrations might also error-prone if one considers cell aggregation, is taking place. Just like a single cell, a cell aggregate delivers only one colony.

Comparing the IMC with the measurement of the optical density in a liquid medium (detection threshold value at 600 nm = 0.01) always an earlier detection (5–9 h) was possible. At low initial bacterial concentrations (<10^2^ CFU⋅mL^–1^), the IMC measurements on solid media were not significantly faster than the classical visual counting of colonies. For the liquid media, in all three measurements, no increase in the optical density was observed for the lowest initial concentration. As already discussed, the existing inoculum volume (10 μL) is too small to ensure the presence of bacteria (see Supplementary Material). It has been proven that the detection time could be greatly reduced by using GF. Here, the detection time for the smallest concentration could be reduced by almost 2 h.

### Reproducibility

**FIGURE 6:**
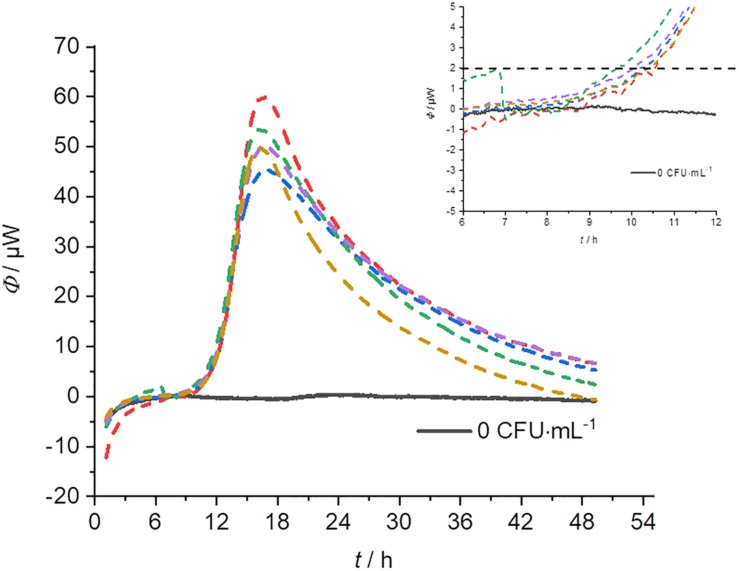
Heat flow signals from *L*. *plantarum* on MRS-agar under identical conditions (*c*_0_ = 10^4^ CFU⋅mL^–1^). The insert provides a magnification of the beginning of the signal which is important for the determination of the detection time.

In order to determine the reproducibility of IMC measurements, five-fold determinations were performed with an identical initial concentration of bacteria for each cultivation technique (except pouring). Figure 6 represents an example of a GOA measurement (for the other measurements see Supplementary Material). Table 3 summarizes the results of these measurements. The error of the detection was roughly similar for GOA, GL and GF. Taking into account the results from Tables 2, 3, it is not surprising that the errors for the filtration technique (GF) were generally slightly higher, because of the difficult handling of the very small filters (*r* = 5 mm). Not every filter could be placed evenly and identically within the measuring ampoule on the nutrient medium. The error caused by the imperfect placement of the membrane filters can potentially be overcome by designing calorimeters that allow the use of standard filters (47 mm).

**TABLE 3:** Summary of the reproducibility measurements of the different cultivation techniques combined with IMC measurments.

**Cultivation technique**	***m* (medium)/g**	***c*_0_/CFU⋅mL^–1^**	***t*_dect._/h**
**GOA**			
1	0.2	10^4^	(10.2 ± 0.3)
2	0.4	10^5^	(7.5 ± 1.1)
**GL**			
1	2	10^7^	(3.6 ± 0.4)
2	2	10^7^	(3.8 ± 0.6)
**GF**			
1	0.4	10^5^	(5.0 ± 0.7)
2^a^	2	10^5^	(4.1 ± 0.4)

*^*a*^Sixfold determination.*

Relative errors in the calorimetric detection time in the same order as for our results were obtained by Bonkat et al. (2013) for bacteria infecting the urinal tract and by Zaharia et al. (2013) for *E*. *coli* and *Staphylococcus aureus*.

## Conclusion

Our data demonstrate that conventional cultivation techniques like pouring (GIA) or plating on agar (GOA), inoculation of broth (GL) or membrane filter cultivation (GF) can be combined with IMC measurements. The great advantage of this technique is the preservation of simplified sample handling and easy evaluation of the data obtained. Only the detector is replaced, i.e., the human eye by a powerful thermoelectric element. In addition, these IMC measurements are non-invasive and non-destructive (Russel et al., 2009; Brueckner et al., 2016). Because the calorimetric experiment measures bacterial growth, an enrichment culture is available after the experiment that can be used for further molecular biological investigations.

It was shown in the case of *E*. *coli* that the detection time in a liquid medium measured by IMC is considerably faster than visual colony counting (Maskow et al., 2012). In order to verify the results, we compared for the first time IMC measurements on solid medium with classical visual colony counting and IMC measurements in liquid medium with classical OD_600_ measurements. We were able to reduce the detection time for both approaches and we were also able to show that enrichment by membrane filtration, in particular, enables a much faster detection time with IMC. When counting the colonies visually, it must be taken into account that further hours elapse before the colony is fully formed and optical identification is possible by morphological properties of the colony. This assumption could be confirmed by the real-time scanning of colony growth (see Supplementary Material). Furthermore, IMC measurements can be used to measure turbid starting solutions and a shaking of the sample is also not necessary.

We were also able to show that in the context of the anaerobic system investigated in this study, the quantity of substrate and the resulting fill level has an influence on the maximum heat flow. GL is preferable to GOA when less sensitive calorimeters are used for the detection of anaerobic microorganisms. However, it has been shown that solid media can also be used for quantification using GIA.

Using selective media, it might be possible to isolate targeted microorganisms from their natural environment and detect them quickly and reliably using IMC (Atlas, 2010). Furthermore, continuous success in the development of selective media for different species in microbiology can be recognized (Bujalance et al., 2006; Nwamaioha and Ibrahim, 2018; Veselá et al., 2019). The exchange of substrates, as well as the addition of antibiotics, enable in some cases the use of a highly selective media and suppressed the growth of accompanying microorganisms. Additionally, the pre-treatment of the sample with chemical treatment like in case of *Legionella* might also suppress the accompanying flora (Bopp et al., 1981). In this way, the lack of specificity of the IMC can potentially be overcome.

The next steps should be the development of simpler and cheaper calorimeters specifically designed for the detection of microbial contaminations in order to become competitive over the conventional approaches (Wadsö et al., 2011) and the investigation of an aerobic system to determine the limitations and applications regarding monitoring bacterial growth by IMC.

Finally, to overcome arbitrary threshold determinations for the heat flow signals mathematical models allowing to discriminate small biological signals from the physical background has to be developed. In this way, a further reduction of the detection time and of the measurement error can be potentially achieved.

## Data Availability Statement

All datasets generated for this study are included in the article/Supplementary Material.

## Author Contributions

TM developed the project idea. CF designed the experimental setup, performed the laboratory work as well as analyzed the data, and edited the manuscript. TM and HH provided consultation for the work and contributed significantly to the preparation of the manuscript. All authors reviewed the manuscript and agreed with the content.

## Conflict of Interest

The authors declare that the research was conducted in the absence of any commercial or financial relationships that could be construed as a potential conflict of interest.

## Footnotes

1 www.tainstruments.com
