# Supplementary material for: Rapid Calorimetric Detection of Bacterial Contamination: Influence of the Cultivation Technique
Source: Front Microbiol. 2019 Nov 1;10:2530. doi: 10.3389/fmicb.2019.02530 (PMC6838224; doi:10.3389/fmicb.2019.02530)
Supplement: Supplementary file 1 [file Data_Sheet_1.docx]

# *Supplementary material*

**Rapid Calorimetric Detection of Bacterial Contamination: Influence of the Cultivation Technique**

**Christian Fricke, Hauke Harms, Thomas Maskow***

Helmholtz-Centre for Environmental Research – UFZ, Department of Environmental Microbiology, Leipzig, Germany

***Correspondence:** Thomas Maskow: thomas.maskow@ufz.de

**Table of contents**

S1: Sample preparation

S2: Baseline correction

S3: CFU-counting results

S4: Heat flow curves of the reproducibility measurements

S5: Reference measurement for the bacterial growth on solid medium using the naked eye for detection

S6: Reference measurement for the bacterial growth on membrane filter using the naked eye for detection

S7: Reference measurements of bacterial growth in liquid medium using OD_600_

# S1 Sample preparation

**Fig. 1** illustrates exemplary prepared glass ampoules with different cultivation approaches before and after an IMC experiment.


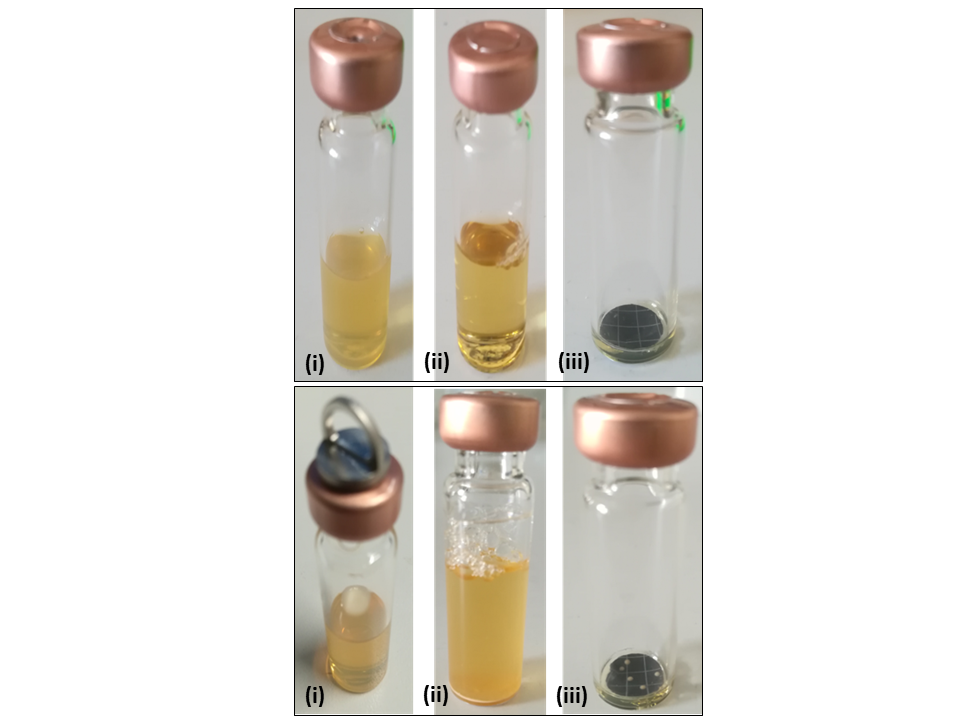


Figure 1: IMC ampoules for each cultivation technique after sample preparation (top) and after an IMC experiment (bottom). i: GOA. ii: GL. iii: GF.

# S2 Baseline correction

**Fig. 2** shows exemplarily the procedure for the baseline correction of a heat flow signal which deviates from the baseline. Using the baseline correction within *OriginPro 2018*, the heat flow signal was corrected.


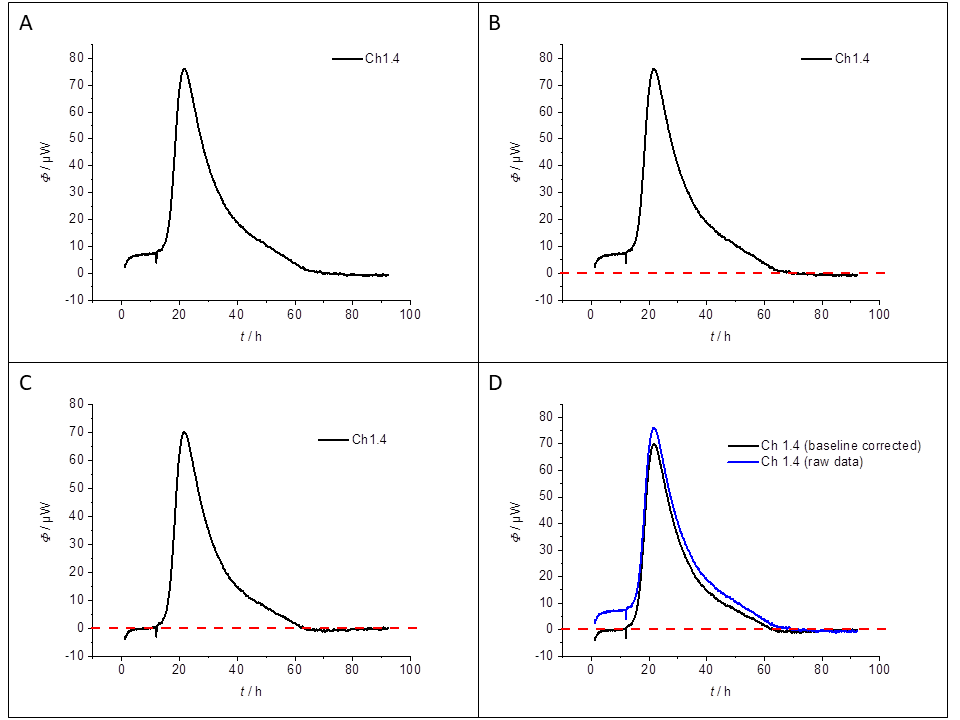


**Figure 2:** Procedure for linear baseline correction by *OriginPro 2018*. **A:** Raw heat flow signal after measurement. **B:** Red dotted line corresponds to the baseline. **C:** The heat flow signal after baseline correction by *OrginPro 2018* (menu option: Analysis $\to$ Peaks and Baseline $\to$ Peak Analyzer $\to$ Subtract Baseline $\to$ Baseline Mode: User defined). **D:** Comparison of heat flow signal before and after baseline correction.

# S3 CFU-Counting results

**Tab. 1** and **Tab. 2** summarize the results of the CFU-counting experiments. The results in **Tab. 1** are corresponding to the experiments for the fill level-dependent and reproducibility measurements as well as for the real-time monitoring after colonies became visible for the eye. The results in **Tab 2.** are corresponding to the investigations of the dependency of the heat traces on the initial bacterial concentration as well as for the real-time monitoring by scanning the plates at defined intervals.

**Tab. 1: Summary of the replicates CFU-countings of fill level-dependent, reproducibility measurements and real-time monitoring by the naked eye.**

| $f_{a}$ | $1$ | 2 | $3$ | $4$ | $5$ | mean | SD |
| --- | --- | --- | --- | --- | --- | --- | --- |
| ${10}^{4}$ | 366400^a^ | 324000^a^ | - | - | - | 345200 | 21200 |
| ${10}^{5}$ | 44400^b^ | 46700^b^ | 50700^b^ | - | - | 47267 | 2603 |
| ${10}^{6}$ | 4300^b^ | 4500^b^ | 4400^b^ | 3130^c^ | 4090^d^ | 4084 | 82 |
| ${10}^{7}$ | 300^b^ | 420^b^ | 360^b^ | 278^e^ | - | 340 | 49 |
| ${10}^{8}$ | 20^b^ | 80^b^ | 90^b^ | 56^e^ | - | 62 | 27 |

^a^OD_600_ was not determined.

^b^OD_600_ = 0.18 (1:100 dilution).

^c^OD_600_ = 0.19 (1:100 dilution).

^d^OD_600_ = 0.20 (1:100 dilution).

^e^OD_600_ = 0.20 (1:100 dilution).

**Tab. 2: Summary of the replicates CFU-countings of the concentration-dependent measurements and real-time monitoring by scanning.**

| $f_{a}$ | $1$^a^ | 2^b^ | $3$^b^ | $4$^b^ | mean | SD |
| --- | --- | --- | --- | --- | --- | --- |
| ${10}^{4}$ | 129000 | 135300 | 121700 | 150200 | 134050 | 10493 |
| ${10}^{5}$ | 15900 | 19400 | 18600 | 13200 | 16775 | 2438 |
| ${10}^{6}$ | 1900 | 1400 | 2200 | 1600 | 1775 | 303 |
| ${10}^{7}$ | 150 | 160 | 180 | 110 | 150 | 26 |
| ${10}^{8}$ | - | 16 | 22 | 10 | 16 | 5 |

^a^OD_600_ = 0.16 (1:100 dilution).

^b^OD_600_ = 0.17 (1:100 dilution).

# S4 Heat flow curves of the reproducibility measurements

**Fig. 3-5** shows for each cultivation technique (GOA, GL and GF) the corresponding heat flow curves.


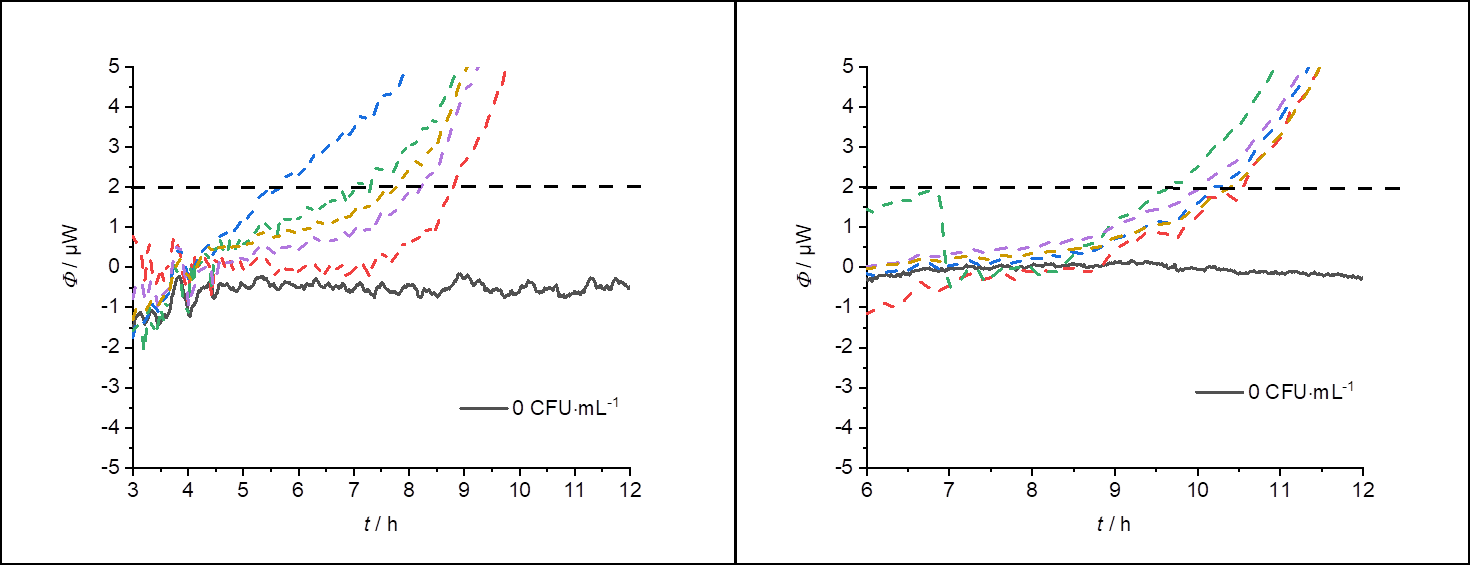


**Figure 3:** Heat flow curves of the reproducibility measurements of GOA.


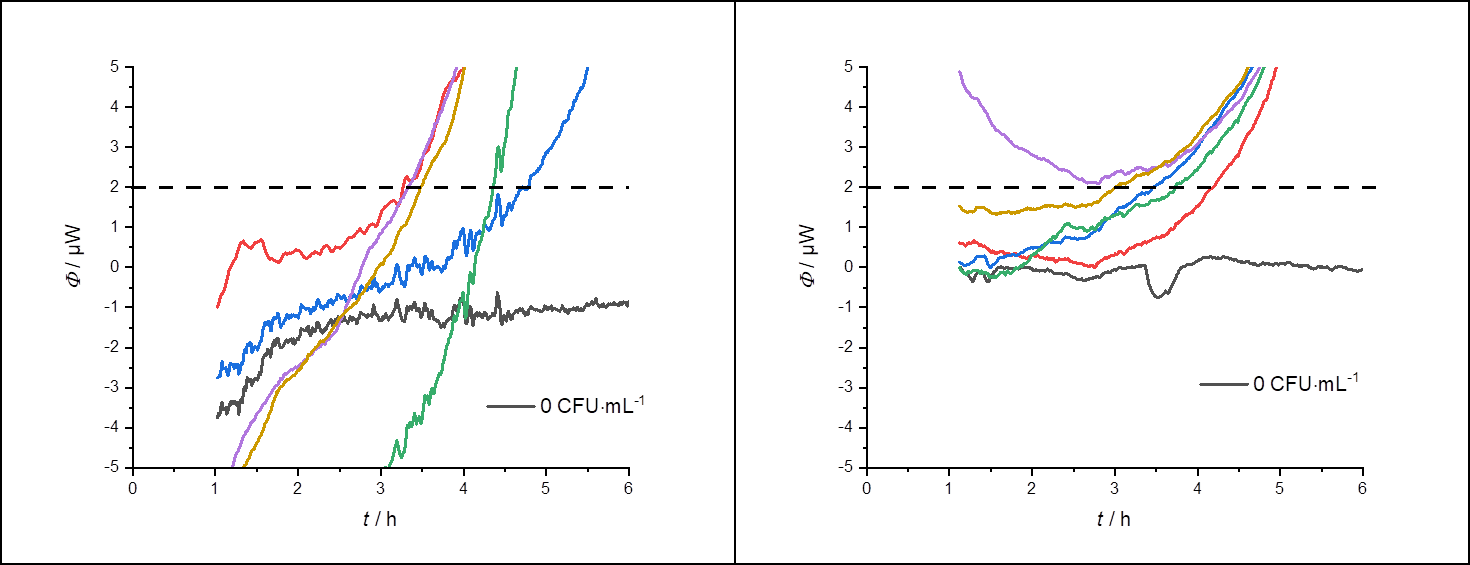


**Figure 4:** Heat flow curves of the reproducibility measurements of GL.


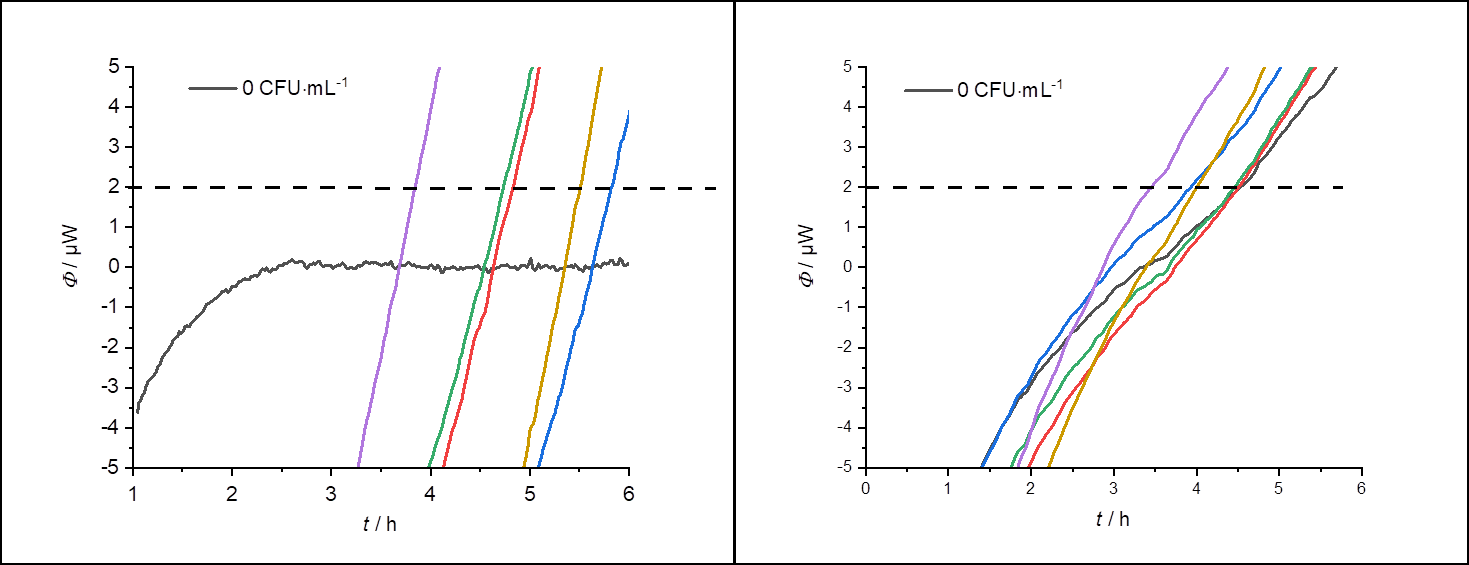


**Figure 5:** Heat flow curves of the reproducibility measurements of GF.

# S5 Reference measurement for the bacterial growth on solid medium using the naked eye for detection

After the experiments, the files were then evaluated. For this purpose, 4 independent persons determined for each dilution level the time from which the colony was visible to the naked eye. The visual detection time was then determined from the results using standard deviation.


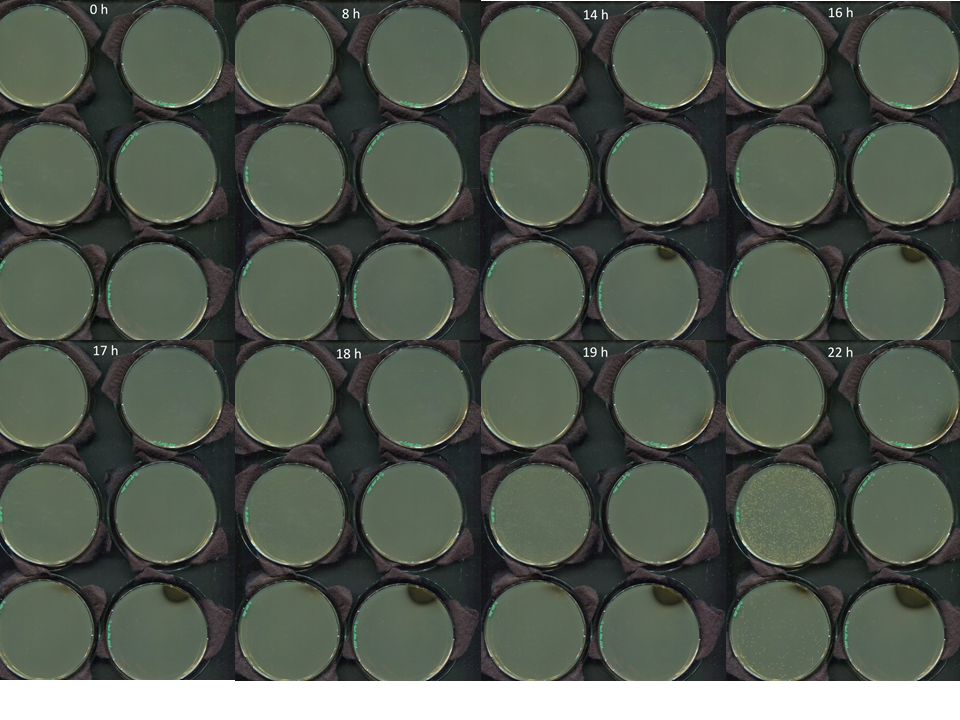


**Figure 6:** Time-dependent recording of the colony forming process on solid media at different initial bacterial concentrations in the time interval from 0 h to 22 h. The detection time $t_{dect.}$ was determined after a colony became visible for the naked eye.

# S6 Reference measurement for the bacterial growth on membrane filter using the naked eye for detection


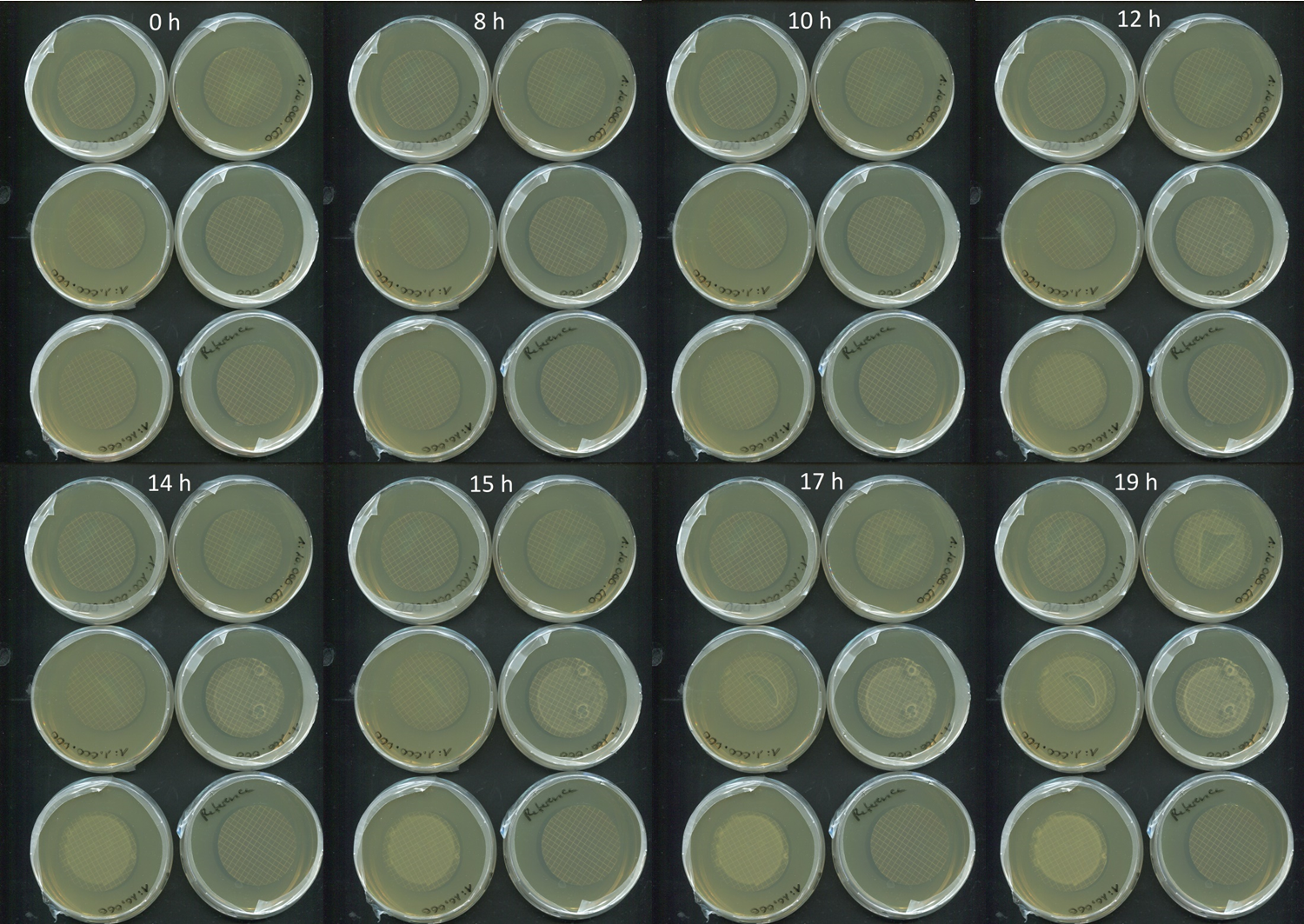


**Figure 7:** Time-dependent recording of the colony forming process on membrane filter at different initial bacterial concentrations in the time interval from 0 h to 22 h. The detection time $t_{dect.}$ was determined after a colony became visible for the naked eye.

# S7 Reference measurements of bacterial growth in liquid medium using OD_600_


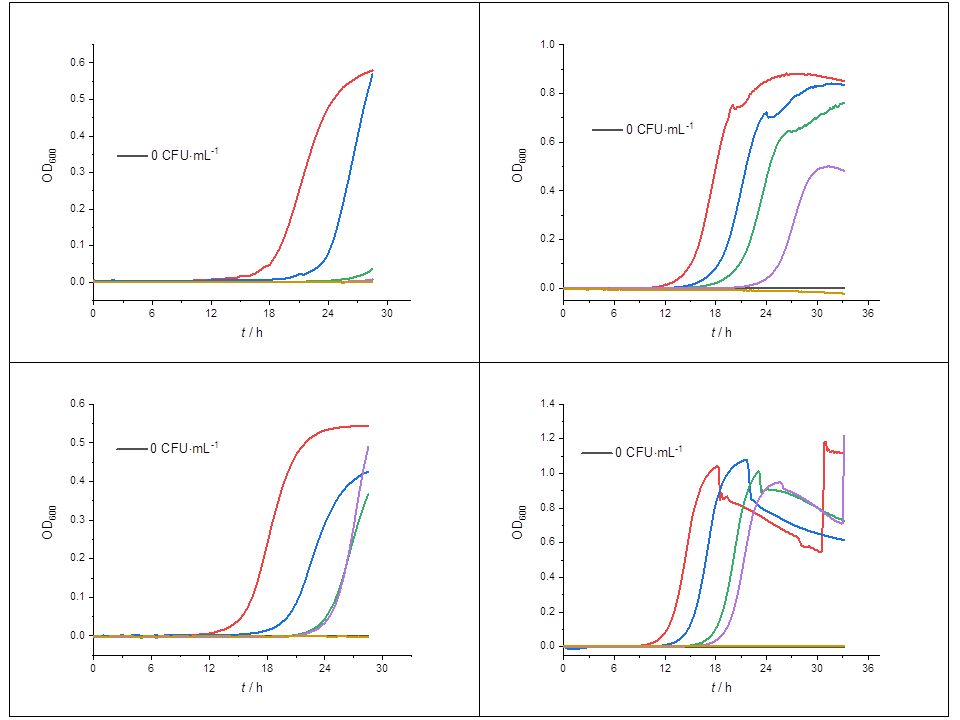


**Figure 8:** Replicates of a time-dependent recording of the OD_600_ at different initial bacterial concentrations under the same conditions. **Red**: 10^5^CFU·mL^-1^. **Blue**: 10^4^CFU·mL^-1^. **Green**: 10^3^ CFU·mL^-1^. **Violett**: 10^2^CFU·mL^-1^. **Yellow**: 10^0^CFU·mL^-1^. **Black**: 0 CFU·mL^-1^. The corresponding detection threshold was set to OD_600_ = 0.01.
